# Ventricular tachycardia revealing drug abuse induced myocarditis: two case reports

**DOI:** 10.1002/ccr3.1542

**Published:** 2018-05-09

**Authors:** Sana Ouali, Omar Guermazi, Manel Ben Halima, Selim Boudiche, Nadim Khedher, Farhati Adeljalil, Fathia Meghaeith, Nourreddine Larbi, Mohamed Samid Mourali

**Affiliations:** ^1^ Cardiology Department Faculté de Médecine de Tunis La Rabta Hospital Université de Tunis El Manar Tunis Tunisie

**Keywords:** Illicit drugs, myocarditis, ventricular tachycardia

## Abstract

Illicit drugs are an uncommon etiology of acute myocarditis but should be evocated in young population. This association may result in further complications, mainly ventricular arrhythmia and therefore increases sudden cardiac deaths among young abusers. Withholding drug intoxication to prevent recurrent events is a major key of management.

## Introduction

Toxicomania is a worldwide health and social problem. In 2015, World Health Organization estimates that 255 millions of people are drug users and more than 10% of them have health disorders 1. Toxicomania induced acute myocarditis is one of the most dangerous reported complications due to its unknown incidence, challenging diagnosis and possible life‐threatening and unpredictable issues as ventricular arrhythmias and heart failure. Here, we present two cases of uncommon acute myocarditis revealed by ventricular tachycardia in the context of multidrug abuse.

## First Case

**Figure 1:**
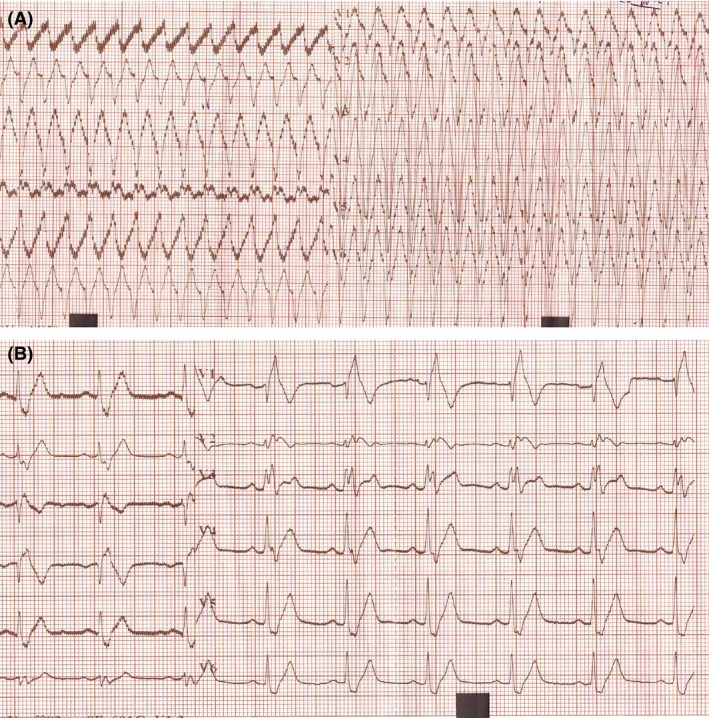
Panel A: ECG in the first patient showing a ventricular tachycardia at 220 beats/min with a negative concordance pattern in all precordial leads and a no man's land QRS axis. Panel B: ECG performed after cardioversion showing a sinus rhythm and a complete right bundle branch block.

**Figure 2:**
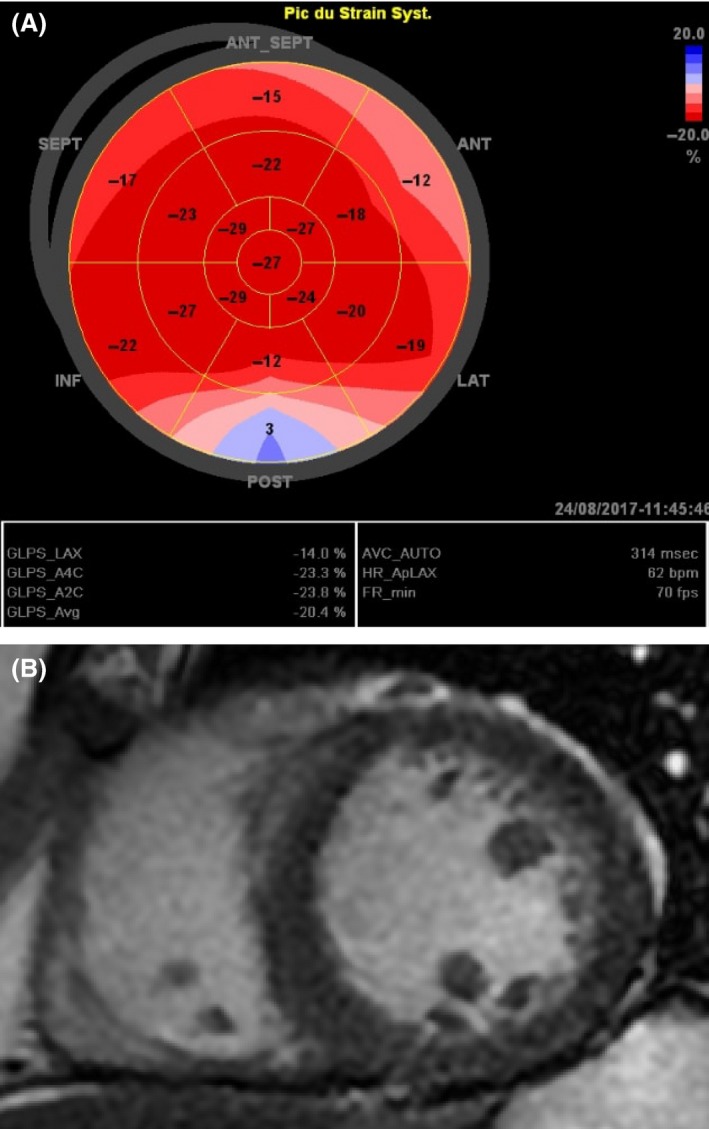
Panel A: Bull's eye strain showing a normal GLS at −20.4%, with a little bit altered in the posterior and postero‐lateral segments of the myocardium. Panel B: Cardiac magnetic resonance imaging (CMR) revealing a LGE of the subepicardial layers of the lateral wall of the left ventricle in patient *n*°1.

A 38‐year‐old man was admitted in our cardiology department for palpitations and dizziness. He was a 27 pack‐years smoker and a multidrug abuser since 10 years. In fact, he was consuming twice a week cocaine and four joints a day of cannabis. An electrocardiogram (ECG) was rapidly performed. It showed a regular wide QRS tachycardia at 220 beats/min with a negative concordance pattern in all precordial leads and an extreme axis deviation (Fig. 1, Panel A). Negative concordance, positive QRS on aVR and R‐wave peak time at lead II (Pava index) at 50 msec were the main criteria to make the diagnosis of ventricular tachycardia (VT). An urgent electrical cardioversion was dedicated by hemodynamic instability (systolic blood pressure at 85 mmHg) and reduced the arrhythmia. Then, the ECG showed a sinus rhythm, a complete right bundle branch block with a QRS duration of 160 msec (Fig. 1, Panel B). Hypersensitive troponins were elevated at 2018 ng/L with normal CPK enzyme. However, metabolic analyses, inflammatory markers as well as coronary angiography were normal. No viral serological studies were performed in admission. A transthoracic echocardiography (TTE) concluded to a normal left ventricular ejection fraction (LVEF) at 60% with normal chest wall motion, a normal right ventricle with no signs of arrhythmogenic right ventricular cardiomyopathy and the absence of valvulopathies. Global longitudinal strain (GLS) was normal at −20.4%, but a little bit altered in the posterior and postero‐lateral segments of the myocardium (Fig. 2, Panel A). In front of these nonconclusive investigations, we performed a cardiac magnetic resonance imaging (CMR) that revealed LVEF of 45% with septal hypokinesia associated with a septal hypersignal with a late gadolinium enhancement (LGE) of the subepicardial layers of lateral wall of the left ventricle (Fig. 2, Panel B). These characteristics were suggestive of myocarditis. Knowing the negative inflammatory markers and the long history of multidrug abuse, the diagnosis of toxic myocarditis revealed by a VT was admitted. We referred our patient to a detoxification center and we prescribed maintaining dose of amiodarone and an angiotensin‐converting enzyme inhibitor until the reversibility of the myocardial damage that we have planned to reevaluate by CMR 6 months after the actual onset. At 2 months of follow‐up, the patient was asymptomatic and he stopped abusing drugs.

## Second Case

**Figure 3:**
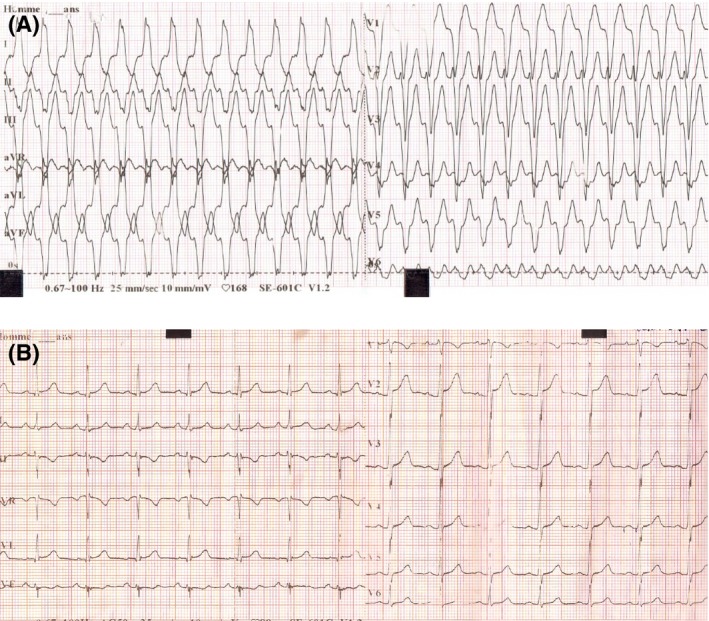
Panel A: ECG in the second patient showing a ventricular tachycardia at 170 beats/min with left bundle branch block pattern and left QRS axis. Panel B: ECG performed after cardioversion showing a sinus rhythm and a narrow but fragmented QRS.

**Figure 4:**
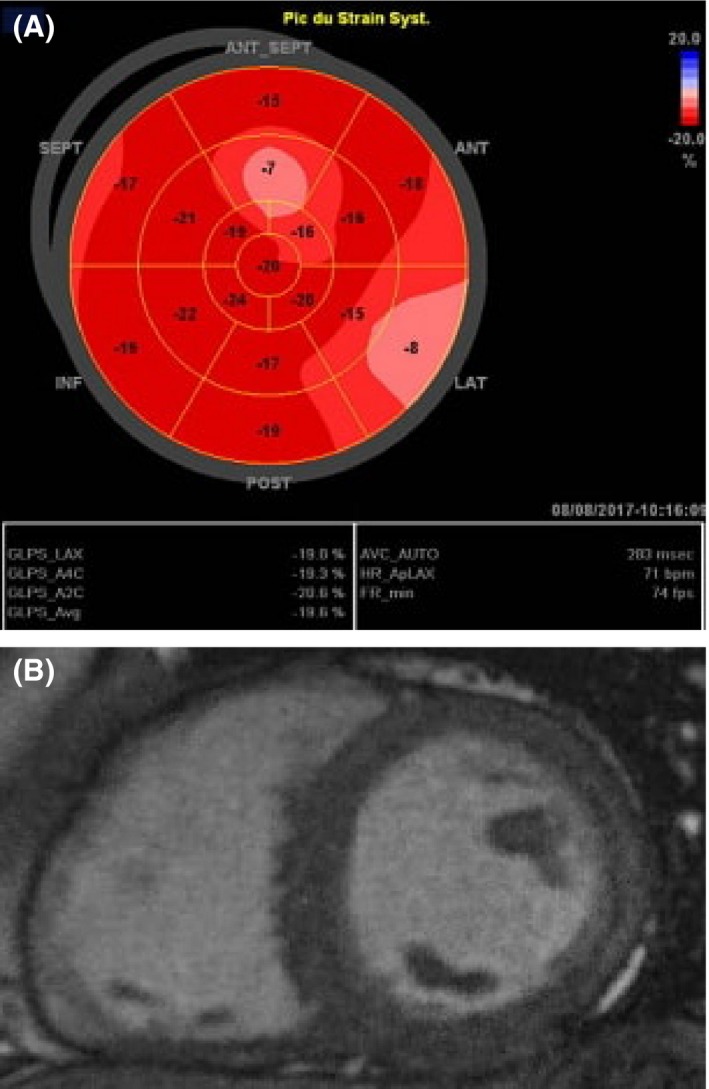
Panel: A Speckle tracking study showing a GLS at −19.6%, a little more altered in the lateral wall of the left ventricle Panel B: CMR revealing a LGE of the lateral wall of the left ventricle in patient *n*°2.

The second case is about a 39‐year‐old man with a history of heavy smoking (50 pack‐years) and a daily three joints of cannabis abuser for 3 years. He is also a monthly cocaine consumer for <1 year. He presented to emergency department for new‐onset palpitations. An immediate ECG demonstrated a regular wide QRS tachycardia at 170 beats/min with left bundle branch block pattern and left QRS axis (Fig. 3, Panel A). More refined interpretation revealed an initial large Q wave on aVR lead, R‐wave peak time at lead II at 70 msec, a positive Vereckei's criterion Vi/Vt (ventricular activation velocity ratio) <1 and an atypical left bundle branch block pattern with R/S ratio <1 on V6 lead. These characteristics were highly suggestive of ventricular tachycardia. Owing to his stable hemodynamic status, pharmacological cardioversion using a loading dose of intravenous amiodarone was attempted. After a successful restoration of sinus rhythm, ECG showed no abnormalities except fragmented narrow QRS complexes on inferior leads (Fig. 3, Panel B). Myocardial enzymes, inflammation markers and ionic analyses, as well as coronary angiography were all normal. No viral serological studies were performed in admission. A TTE demonstrated a normal global and regional left ventricular function with a LVEF at 60%, without right ventricular or valve anomalies. Speckle tracking study showed a GLS at −19.6%, a little more altered in the lateral wall of the left ventricle (Fig. 4, Panel A). In the sight of this VT with apparent normal heart, CMR study was obtained within 48 h of presentation. It revealed moderately reduced LVEF at 48% with lateral hypokinesia and spontaneous T2 hypersignal of the subepicardial layers of the lateral left ventricular wall corresponding to the areas of acute edema and inflammation. LGE images showed linear subepicardial enhancement of the lateral wall of the left ventricle (Fig. 4, Panel B). Hence, a drug abuse induced acute toxic myocarditis was the most likely diagnosis. As in first case, we referred our patient to a detoxification center and we prescribed amiodarone in order to prevent arrhythmia recurrence and an angiotensin‐converting enzyme inhibitor to avoid further ventricular remodeling. CMR was planned 6 months later in order to control the reversibility of myocardial damage. Our patient has been just reexamined. He definitely withheld toxicomania and begun a smoking cessation plan. He remained asymptomatic and his ECG unchanged.

## Discussion

According to Lake Louise consensus criteria, CMR findings suggestive of myocarditis are as follows: (1) Regional or global myocardial signaling intensity increase in T2‐weighted images suggestive of edema; (2) Increased global myocardial early gadolinium enhancement ratio between myocardium and skeletal muscle in gadolinium‐enhanced T1‐weighted images suggestive of hyperemia and capillary leakage; (3) At least one focal lesion with nonischemic regional distribution in inversion recovery prepared gadolinium‐enhanced T1‐weighted images (late gadolinium enhancement) suggestive of necrosis and fibrosis. In the setting of clinically suspected myocarditis, CMR findings are consistent with myocardial inflammation, if at least two of the above‐cited criteria are present 2. If all sequences can be performed and two or more of the three tissue‐based criteria are positive, myocardial inflammation can be predicted or ruled out with a diagnostic accuracy of 78%, and if only LGE imaging is performed, the diagnostic accuracy is 68% 2. Our two patients fulfilled at least the first and third criteria. Hence, it was contributive to the diagnosis. Speckle tracking is a new echocardiographic tool that may contribute to the diagnosis of myocarditis when LGS is altered with a sensitivity of 78%, a specificity of 93% and an optimal cutoff value of ‐15.1% 3. In our reported cases, LGS was normal maybe because of the limited necrosis area as shown later by CMR.

In a series of 40 autopsies performed in the context of cocaine‐associated deaths, it was demonstrated that myocarditis with mononuclear infiltrate was responsible of 20% of deaths and was nearly 10 times more common than acute thrombotic coronary occlusion.

This finding showed that drug abuse induced myocarditis could be responsible of sudden cardiac death likely via ventricular arrhythmia, which was the relevant complication in our two patients.

The evocated mechanism of VT, in this case, is scar‐related macroreentry due to the development of necrosis and fibrosis as shown by CMR 4. Moreover, catecholamine excess induced by cocaine or cannabis intoxication can also be evocated as VT trigger on such a vulnerable myocardium 5, 6.

In our both cases, the VT morphology on ECG have suggested an apical VT exit; however, myocardial scars on CMR were specially located in the subepicardial layers of lateral wall of the left ventricle.

In the first case, cocaine seems to be the etiology of the myocardial damage owing to the more established association between cocaine and myocarditis and also the existence of ECG signs of cocaine intoxication revealed after the reduction of VT. In fact, complete right bundle branch block, shown in our case, was described to be the consequence of sodium channel blockade, the main mechanism of action of cocaine 5. In contrast, for the second patient, cannabis is likely the responsible drug because of the occasional consumption of cocaine compared to daily use of cannabis, in addition to the absence of cocaine‐related ECG signs 7. However, cannabis is uncommonly reported to be associated with myocarditis 8, 9. The presence of adulterants or contaminants (such as solvents and pesticides) in manufactured modified cannabis could be a possible mechanism responsible for the cardiotoxic effects of cannabis 10. Nonetheless, to our knowledge, there is no published literature that mentions the histopathological alterations induced by cannabis (autopsy series).

In both cases, the diagnosis of drug‐induced myocarditis was a diagnosis of exclusion. Autoimmune and viral myocarditis should be mentioned. Nonetheless, given the history of the two patients and the electrocardiographic pattern in the first case, in relation with cocaine and or Marijuana should raise suspicion for a causal association. Performing an endomyocardial biopsy could help the etiological diagnosis, since in drug abuse myocarditis, contraction band necrosis could be demonstrated and could confirm the causal association 7.

In our patients, we preferred not to prescribe beta blockers as it is contraindicated in case of cocaine intoxication because of the risk of coronary vasospasm and myocardial infarction 11. For ventricular tachycardia, implantable cardioverter defibrillator implantation should be deferred until resolution of the acute episode. Because of beta blockers were contraindicated in this particular situation, the choice of amiodarone was indicated.

## Conclusion

Ventricular tachycardia is a challenging finding, in which CMR plays a key role for the diagnosis of potential reversible causes mainly acute myocarditis. This complicated situation can be induced by illicit drug abuse. Such an uncommon association should notably be evocated in young population and must lead to definitively stop abusing the culprit drug.

## Authorship

SO, OG: conceived of this case report. All authors read and approved the final manuscript.

## Conflict of Interest

None declared.
